# Adenoid Disease in School Children

**Published:** 1913-04-19

**Authors:** Thomas Dutton

**Affiliations:** 25 New Cavendish Street, Harley Street, W.


### THE EDITOR'S LETTER-BOX

### Adenoid Disease in School Children.

*To the Editor of* The Hospital.

Sir,—It was refreshing to read the article on this subject in The Hospital of March 15, p. 643. It shows the writer to be one of the few conscientious, impartial practitioners of the present day. I fully endorse, from long clinical experience, the following : "To advise operation in every case in which a child is a mouth breather, or in which the tonsils are enlarged, is to court indifference and neglect of all subsequent advice." I find that among the better-class children operating for adenoids or enlarged tonsils is rarely required, if the parents will carry out treatment for a prolonged period—viz., breathing exercises, a wide band to close the mouth during sleep, local applications of stringent lotions, careful dieting, and regular visits to the doctor.

I am firmly convinced of one thing—that is a child who is cured in this manner is far stronger than a child who has been operated upon. The tonsils have no doubt most important functions to fulfil, and the adenoids should never be allowed to grow to require a knife to remove them.

I have no objection to an operation if the child has been under the above treatment without any improvement for, say, two months.—I am, Sir, yours truly,

Thomas Dutton, M.D.

25 New Cavendish Street, Harley Street, W.,
April 15, 1913.

